# Access to and Quality of Neighbourhood Public Open Space and Children’s Mental Health Outcomes: Evidence from Population Linked Data across Eight Australian Capital Cities

**DOI:** 10.3390/ijerph19116780

**Published:** 2022-06-01

**Authors:** Amanda Alderton, Meredith O’Connor, Hannah Badland, Lucy Gunn, Claire Boulangé, Karen Villanueva

**Affiliations:** 1 Centre for Urban Research, RMIT University, Melbourne 3000, Australia; hannah.badland@rmit.edu.au (H.B.); lucy.gunn@rmit.edu.au (L.G.); claire.boulange@rmit.edu.au (C.B.); karen.villanueva@mcri.edu.au (K.V.); 2 Murdoch Children’s Research Institute, Royal Children’s Hospital, Melbourne 3052, Australia; meredith.oconnor@mcri.edu.au; 3 Department of Paediatrics, University of Melbourne, Melbourne 3052, Australia

**Keywords:** mental health, child development, inequities, social determinants, built environment, green space, public open space

## Abstract

Neighbourhood-level interventions offer a promising opportunity to promote child mental health at a population level; however, neighbourhood effects are still regarded as a ‘black box’ and a better understanding of the specific design elements, such as public open space, is needed to inform actionable policy interventions. Methods: This study leveraged data from a population linked dataset (Australian Early Development Census—Built Environment) combining information from a national census of children’s developmental outcomes with individualised geospatial data. Associations between access to (within 400 m and 800 m from home), and quality of, public open space and child mental health outcomes across eight capital cities were estimated using multilevel logistic regression models, adjusting for demographic and contextual factors. Access was defined based on proximity of public open space to children’s home addresses, within distance thresholds (400 m, 800 m) measured along the road network. Effect modification was tested across maternal education groups. Results: Across the eight capital cities, inequities in access to child friendly public open spaces were observed across maternal education groups and neighbourhood disadvantage quintiles. Children with access to any type of public open space within 800 m of home had lower odds of demonstrating difficulties and higher odds of competence. Children with access to child friendly public open spaces within 800 m of home had the highest likelihood of demonstrating competence. Conclusion: Improving access to neighbourhood public open space appears to be a promising strategy for preventing mental health difficulties and promoting competence in early childhood. Action is needed to redress socio-spatial inequities in access to child friendly public open space.

## 1. Introduction

Mental health difficulties affect between 10% to 20% of children globally [1,2]. For many children, these difficulties first emerge in the early years [3], during which the brain is highly sensitive to nurturing environments and stimulating interactions with caregivers [4]. Young children’s mental health during the first decade of life forms a foundation for their mental health and social functioning throughout the life course, and is therefore critical to strategies aimed at promoting mental health at a population level [5].

In the early years, mental health difficulties are often conceptualised according to externalising difficulties (difficulties with behaviour or inattention) and internalising difficulties (difficulties with emotions such as anxiousness, fear, or sadness) [6]. Mental health competence (i.e., positive mental health) in early childhood includes psycho-social skills such as regulating emotions and behaviour, skills in interacting with peers, and caring for others [7,8]. Evidence suggests that both mental health difficulties and competence carry implications for important health and social outcomes, such as young children’s capacity to engage with early learning experiences [9,10,11].

Bio-ecological models recognise that children’s mental health is influenced by multiple contexts in which children develop, including the neighbourhood, as well as the family and wider societal contexts [12]. It has long been observed that neighbourhoods matter to young children’s mental health, and this is well-documented in the ‘neighbourhood effects’ literature [13,14]. For example, children growing up in disadvantaged neighbourhoods are more likely to demonstrate mental health difficulties—irrespective of their individual family circumstances—compared with their peers in more advantaged neighbourhoods [15]. However, these neighbourhood effects are still largely regarded as a ‘black box’, prompting calls for a deeper understanding of the specific neighbourhood-level factors—especially those that are modifiable by policy and planning interventions—that support better mental health in early childhood [14]. Such identification of specific, modifiable neighbourhood features could enable a promising opportunity to promote child mental health at a population level, as neighbourhood-level interventions have the benefit of reaching large numbers of children and their families.

One key feature of a supportive neighbourhood for children is the availability and quality of neighbourhood public open space (e.g., parks, playgrounds). High-quality public open space provides opportunities for play and stimulating activities, socialisation, and skill-building (e.g., trying out new experiences, engaging with other children) [16]. It seems likely that these experiences play a role in promoting children’s mental health. Qualitative research with young children’s caregivers points to public open space as a key community asset that families regard as important for young children’s development and skill-building [17]. It has also been theorised that ‘green space’—public open space with natural features (e.g., trees and vegetation)—may have a psychologically restorative or protective effect, helping to reduce or prevent mental distress [18]. In older children, several studies have found a protective relationship between exposure to green space and reduced mental health difficulties, particularly hyperactivity and inattention [19,20,21,22]. Not all parks are created equal, however, with some evidence indicating that the quality of public open space makes a difference; for example, some studies have found more consistent or larger magnitude of associations between young children’s mental health and measures of quality of public open space, versus measures of quantity or proximity [23,24,25]. From a child- and family-centred perspective, high-quality public open space includes features supporting play and recreation (e.g., playgrounds) as well as facilities that enable families to use the space over the course of several hours (e.g., public toilets) [17].

Despite the plausibility of the relationship between early childhood mental health and public open space access and quality, the evidence base is nascent. Recent systematic reviews of the evidence around public open space and child mental health have identified a lack of studies investigating associations with mental health in the early childhood period, and mental health competence in particular [26,27]. Furthermore, very few studies have examined the quality of public open space and how this relates to young children’s mental health [26,27]. This gap exists despite a growing body of evidence suggesting the importance of public open space quality, in particular, for promoting mental health. For example, a study conducted with adults in Australia found that those with access to high-quality public open space were around two times more likely to have low psychological distress than those with access to low-quality public open space [28]. The authors of another study found that positive mental health was associated with specific quality features (natural areas, sporting, recreation) of public open space, with the largest effect size observed for sporting features [29].

In addition to these gaps, a major limitation of the current evidence is a lack of studies attempting to unpack how specific neighbourhood attributes relate to children’s experiences of disadvantage [26]. Inequity between neighbourhoods in terms of the resources and opportunities provided to young children and families has been postulated as a potential mechanism through which neighbourhood effects on children’s early mental health are transmitted, with children living in the most disadvantaged neighbourhoods having fewer opportunities to build social and emotional skills [14,30]. Internationally, studies examining the relationships between neighbourhood disadvantage and public open space availability and quality have reported somewhat mixed findings across different countries and contexts, but there is some suggestion that those living in the most disadvantaged neighbourhoods may have worse access to, and poorer quality of, local public open space than their peers in more affluent neighbourhoods [31,32,33]. Research examining how access and quality of neighbourhood public open space relates to young children’s experiences of disadvantage is urgently needed, given evidence that children facing disadvantage experience a double burden of higher mental health difficulties and lower mental health competence compared with their more advantaged peers [34,35,36].

Data linkage offers new ways of answering these questions and clarifying associations across large numbers of children, and potentially entire populations of children. Previous data linkage of early child development outcomes and built environment measures have successfully generated evidence for individual Australian cities [24,37]. The present study extends this work by leveraging a globally unique population linked dataset combining geographic and objective data on children’s neighbourhood-built environments with data collected as part of the Australian Early Development Census (AEDC), a population census of early child development, across Australia’s eight capital cities. The aim of this study is to: (1) document access to public open space in a national cohort of children, and describe how this access varies between relatively socioeconomically advantaged and disadvantaged children, (2) examine relationships between access to and quality of neighbourhood public open space and mental health (externalising difficulties, internalising difficulties, and competence) in early childhood, and (3) investigate whether these relationships differ among children by socioeconomic disadvantage. In doing so, we aimed to inform urban planning and policy, as well as early childhood and education portfolios, by developing the evidence base around what neighbourhood characteristics can be modified at scale to support mental health in the early years.

## 2. Materials and Methods

### 2.1. Study Design: Australian Early Development Census—Built Environment (AEDC-BE)

This study uses a cross-sectional population linked dataset, the Australian Early Development Census—Built Environment (AEDC-BE) dataset, which combines (1) child demographics and developmental outcomes, collected as part of the Australian Early Development Census (AEDC) in 2015, (2) geographic data (e.g., administrative boundaries, city and state of residence), and (3) objective measurements of children’s neighbourhood built environments; we provide a detailed description of this data in another article of this special issue [38]. Details of each of these component datasets and data linkage procedures are described further below.

### 2.2. Australian Early Development Census

Every three years, the Australian Government funds a population census of children’s early development, the Australian Early Development Census (AEDC). The AEDC provides a snapshot of child development for all children across Australia who are entering their first year of full-time schooling. Teachers complete the instrument, an adapted version of the Canadian Early Development Index [39], for each child in their class, responding to a range of items encompassing physical health and wellbeing, social competence, emotional maturity, language, cognitive skills (school-based), communication skills, and general knowledge [40]. The AEDC’s exceptional population coverage (including 96.5% of school entrants in 2015, with a school participation rate of 96.7%) is achieved through a range of strategies, such as its use of secure web-based data entry system and funding to cover teacher relief (covering each teacher for one hour of training and 20 min per instrument) [41,42].

### 2.3. Geographic and Neighbourhood Built Environment Data

Objective neighbourhood-built environment measures were developed for Australia’s most populous 21 cities (as in Australia’s National Cities Performance Framework) [43]. These measures formed part of a pre-existing database used to calculate national parcel-level liveability indicators across Australia [44]. These measures, along with geospatially referenced 2016 Australian Census data, were linked to unique home addresses of children from the 2015 AEDC. This process involved matching children’s home addresses to the nearest sampling point location in the national database (average distance from child’s home address to nearest sampling point for this capital cities child cohort: 3.5 m). Sampling point locations, which served as proxies for residential lots, were derived from the Geocoded National Address File, Australia’s authoritative national address database produced and maintained by Geoscape Australia (formerly PSMA) [44,45,46]. A detailed description of sampling point methodology is presented elsewhere [44,45].

Over 80 spatial measures of the neighbourhood environment were calculated for each child, including information about the public open space available around the child’s home and the attributes of these spaces. These measures were developed and conceptualised as part of the child liveability work programs in the Centre for Urban Research at RMIT University and Murdoch Children’s Research Institute [38,47,48]. Given that these spatial measures were conceptualised for urban areas, children living in remote or very remote locations were not included in the linked AEDC-BE dataset.

### 2.4. Study Participants

In total, 302,003 children participated in the AEDC in 2015, of which 235,631 children resided in 21 of Australia’s most populous cities and towns and were included in the AEDC-BE linked dataset. The present analysis focuses on Australia’s capital cities’ school entrant population, including 199,200 children (approximately two of every three children nationally) living in Adelaide, Brisbane, Canberra, Darwin, Hobart, Melbourne, Perth, and Sydney. We focused on capital cities as a starting point for building this evidence base, because we expected that relationships with mental health might differ in rural and regional towns and require focused exploration. Australia’s eight capital cities are diverse, yet they are unified in that they represent the major metropolitan centre of each state or territory.

### 2.5. Data Linkage Procedures

The data linkage procedures and geospatial measures included in the AEDC-BE dataset are presented in detail elsewhere [38,48]. Briefly, the AEDC data custodians, the Social Research Centre (SRC) (on behalf of the Australian Government Department of Education and Training), provided geocoded addresses (latitude and longitude coordinates) of 2015 AEDC participants as well as child demographics and development data to the Australian Institute of Family Studies (AIFS), an approved data linkage body. AIFS provided RMIT University with a de-identified AEDC participant list of geocoded addresses, including an additional 5% of false addresses to help ensure anonymity. The spatial measures of children’s neighbourhood environment were calculated and attached to the participant list and returned to AIFS, who removed the false addresses, de-identified the final linked dataset by removing the geocoded addresses and integrated the spatial neighbourhood environment measures with AEDC content data (e.g., demographics and child development outcomes). The final de-identified linked dataset was then provided to the research team for analysis. Data linkage was undertaken by AIFS in August 2019.

### 2.6. Mental Health (Outcome) Measures

Measures of each mental health outcome (internalising difficulties, externalising difficulties, mental health competence) have been previously conceptualised and derived from the AEDC. These indicators are developmentally appropriate for early childhood and have been validated against the Strengths and Difficulties Questionnaire [7,10,35,36,49]. Teachers reported on children’s demonstration of behaviours and skills using Likert scales (e.g., ratings of very good/good, average, and poor/very poor) or binary (yes/no) scales. For each mental health outcome, the average score of the relevant AEDC subdomains (detailed below) was calculated for each child. As is the case for other domains of the AEDC, all three of the outcomes examined here had a strongly skewed distribution and were therefore dichotomized, as is recommended for other AEDC domains [40]. Cut-points for dichotomisation used the bottom tertile for difficulties or competencies following previous research [10], and were the same as the cut-points based on the distribution of scores in the entire AEDC-BE national cohort (i.e., 21 most populous cities), facilitating future comparisons between the eight capital cities of focus in this study and the broader urban child population in Australia.

#### 2.6.1. Externalising Difficulties

Externalising difficulties were indicated by teacher reports on the AEDC subdomains of hyperactivity and inattentiveness (e.g., cannot settle to anything for a few moments) and aggressive behaviours (e.g., gets into physical fights). This measure was dichotomised using the first tertile as a cut point, with children in the poorest scoring tertile being classified as demonstrating ‘high’ difficulties (as compared with the remaining children, who were classified as demonstrating ‘low’ difficulties).

#### 2.6.2. Internalising Difficulties

Internalising difficulties were indicated by teacher-reported items from the anxious or fearful behaviours (e.g., is nervous, highly strung or tense) AEDC subdomain [10]. This measure was dichotomized into ‘high’ and ‘low’ difficulties in the same way as externalising difficulties.

#### 2.6.3. Competence

The competence measure includes teacher reported skills of overall social competence (e.g., ability to get along with peers), approaches to learning (e.g., works independently), readiness to explore new things (e.g., is eager to play with a new toy), prosocial and helping behaviour (e.g., will try to help someone who is hurt). This measure was dichotomised at the top tertile, such that children with the most exceptional strengths were classified as demonstrating ‘high’ competence (as compared with the remaining children, who were classified as demonstrating ‘low-to-moderate’ competence).

### 2.7. Public Open Space (Exposure) Measures

Data for the public open space measures in the AEDC-BE dataset were sourced from OpenStreetMap in 2018 [50]. OpenStreetMap is a community contributed, open-source database cataloguing spatial neighbourhood data [50]. A major strength is its consistency across the eight capital cities, given that at the time of data linkage there was not an existing national database of public open space in Australia. Detailed information on the types of land uses and space included and excluded in the study’s definitions of public open space, and the operationalization of these using OpenStreetMap, are presented in Supplementary Materials (Table S1), and additional metadata can be found in the Australian Urban Observatory [51].

Each of the public open space access measures were derived from continuous variables measuring the distance, in metres along the road network, from a child’s home to the nearest (a) ‘child friendly’ public open space with both a playground and a toilet nearby (i.e., within 100 m of the public open space boundary) or (b) public open space of other types, but lacking these child friendly features (hereafter ‘non-child friendly’ public open space). Detailed information about the construction of these spatial measures is presented in the Supplementary Materials (Table S1) and developed following guidance from Lamb and colleagues [52]. The definition of child friendly public open space was based on qualitative research with caregivers of young children across Australia, which pointed to the salience of these two quality features as especially important for supporting young families’ use of public open space [17]. To facilitate their interpretation in terms of tangible policies and interventions, public open space measures were categorised into three mutually exclusive groups (no access; access to non-child friendly public open space; access to child friendly public open space) to indicate both access and quality within two distance thresholds: 400 metres and 800 metres. These distances were chosen based on the previous child health literature [27] and current Australian policy standards for public open space provision [53,54]. For example, although little is known about the size of the areas within their neighbourhoods where young children roam, previous Australian research suggests that on average, caregivers perceive 1.5 km (approximately 30 min of walking) to be an appropriate walking distance for young children [55], which aligns closely with an 800 m one-way (1600 m round-trip) walking distance.

### 2.8. Demographic (Covariate) Measures

Demographic measures were drawn from the AEDC, almost all of which were pre-populated from information collected through school enrolment processes, except for one teacher-reported item assessing English proficiency. These measures represent markers of exposure to structural inequities, and therefore, are likely to be associated with both exposure (neighbourhoods in which children live) and mental health outcomes.

#### 2.8.1. Maternal Education

Maternal education was based on the highest level of schooling and highest post-school qualification by the caregiver listed as ‘Parent 1’ for the child. Australian data shows that ‘Parent 1’ is almost always the child’s biological mother [56,57]. Three groups were derived to cover high, middle, and low maternal education groups in the Australian context: (1) bachelor’s degree or higher, (2) completed high school (Year 12) and/or other tertiary post-school qualification(s) (e.g., trade qualifications), (3) did not complete high school (Year 11 or less).

#### 2.8.2. Sex

A dichotomous (male, female) measure of the child’s sex was included to account for any sex-specific differences in mental health.

#### 2.8.3. Indigenous Status

A dichotomous (non-Indigenous, Aboriginal, and Torres Strait Islander) measure was used. The aim of including this measure was to factor into analysis the historical and contemporary impacts of colonization and systemic racism that children and families from Indigenous backgrounds in Australia continue to face, but which is rarely captured in administrative data sources [58].

#### 2.8.4. Language Background and English Proficiency

This study used a measure of language background and English proficiency de-veloped by Goldfeld and colleagues [59], which was based on one pre-populated item from the AEDC indicating language background other than English (yes/no) and one teacher-reported item assessing English proficiency of the child. From these two pieces of information, three groups were derived: (1) English only (any proficiency), (2) multilingual (English-proficient), (3) multilingual (English-emerging). English-proficient was defined as any child who was rated by their teacher as having average or good-to-very good English proficiency. This measure was included in the analysis to account for the marginalisation and social exclusion that is often experienced by children and families from diverse cultural and linguistic backgrounds, including in the classroom, given English is the dominant language of instruction in Australia.

#### 2.8.5. Additional Health and Education Needs

A dichotomous measure indicating whether the child was identified as having additional health and education needs was included. Children were identified as having additional health and education needs if they had a medical diagnosis of a chronic medical, physical, or intellectually disabling condition that requires special assistance [60]. This measure was included to account for the barriers these children face in participating in the opportunities that build mental health, which result in higher risk of mental health difficulties.

### 2.9. Contextual (Covariate, Study Area, and Adjustment for Clustering) Measures

#### 2.9.1. Neighbourhood Boundaries

Neighbourhood geographic boundaries were approximated in this study using the Australian Bureau of Statistics Statistical Area 1 (SA1), which represents a small area with a population size of approximately 400 people [61].

#### 2.9.2. Neighbourhood Disadvantage

Neighbourhood disadvantage was measured at the SA1 using the Australian Bureau of Statistics Socio-Economic Index for Areas—Index of Relative Socio-Economic Disadvantage (SEIFA-IRSD) [62]. This commonly used multidimensional index captures multiple aspects of an area’s disadvantage, such as proportion of residents on low incomes, proportion of residents without internet access [62]. Neighbourhood disadvantage quintiles were calculated by the Australian Bureau of Statistics for all SA1s in Australia, prior to data linkage; hence, neighbourhood disadvantage quintiles represent the socioeconomic characteristics of a neighbourhood relative to all other neighbourhoods across Australia. The use of quantiles (rather than raw scores) is recommended by the Australian Bureau of Statistics [62] and SEIFA-IRSD quintiles are commonly reported on and used in analyses of data from the AEDC e.g., [24,35,60,63].

#### 2.9.3. Capital City of Residence

Capital city boundaries were defined using the Australian Bureau of Statistics Greater Capital City Statistical Areas (GCCSAs), which are intended to capture the functional extent of a capital city [61]. Rather than capturing the built-up edge of the city, each GCCSA reflects a more nuanced understanding of urban areas, including people who live in nearby towns but regularly work, shop or visit the capital city [61].

### 2.10. Statistical Analysis

First, a descriptive picture of the entire capital cities cohort was generated, including cohort characteristics expressed as a percentage (e.g., percentage female) as well as the characteristics of children with: (a) no access to public open space, (b) access to non-child friendly public open space, and (c) access to child friendly public open space. Next, within each of the three maternal education groups, the percentages of children in each public open space exposure level (no access, non-child friendly public open space, child friendly public open space) were calculated and displayed visually. These percentages were also calculated and graphed separately within each of the neighbourhood disadvantage quintiles. Descriptive statistics were calculated in Stata 16 [64].

Statistical analysis was informed by a directed acyclic graph, presented in Supplementary Materials (Figure S1). A multilevel modelling approach was used to account for the non-independence (i.e., clustering) of children’s mental health outcomes within neighbourhoods (i.e., SA1s). First, univariate, multilevel (i.e., children nested in neighbourhoods) logistic regression models (Model A) were used to estimate the unadjusted association between each public open space exposure measure and the odds of each mental health outcome. These associations were modelled separately for each of the four exposures and by each of the three outcomes, resulting in a total of 12 models.

Multivariable multilevel logistic regression models were used to estimate the adjusted associations. Based on the directed acyclic graph, we identified two sets of adjustment variables aligning to two different (but not mutually exclusive) assumptions about the underlying causal pathways between neighbourhood disadvantage, public open space access by type, and children’s mental health: the first assumption is that there is systematic under-investment in public open space in disadvantaged neighbourhoods; alternatively, the second assumption is that provision of public open space drives up property values, leading to gentrification, displacement of disadvantaged residents, and reduction in neighbourhood disadvantage. The first set of adjustment variables included child- and family-level demographic characteristics only (Model B); the second included these demographic characteristics as well as neighbourhood disadvantage (Model C). Both adjustment sets were modelled.

All models were estimated using Markov Chain Monte Carlo estimation procedures using MLwiN and Stata 16’s runmlwin command [65,66]. Model convergence was checked by visually examining trace plots generated in MLwiN. For Models A and B, a chain length of 35,000 and burn-in of 2000 iterations was used. Model C was run with a burn-in of 5000 iterations and chain lengths of 35,000 (no interaction terms) and 50,000 (Model C plus interaction terms).

### 2.11. Sensitivity Analysis

To assess whether associations were different within each capital city, we aimed to undertake a sensitivity analysis. Models A (unadjusted), B (adjusted for child- and family-level characteristics), and C (Model B adjustments, plus neighbourhood disadvantage) were run using the same procedures outlined above, with children stratified by capital city; however, the stratified results were not able to be reliably estimated due to issues with model convergence for Models B and C. Hence, we present results for the entire capital cities cohort and can only speculate on city-specific associations with full adjustment.

### 2.12. Missing Data

AEDC has exceptional population coverage, with only 1% missing data on average across study variables included in this analysis; hence, analysis focused on children with complete data across all study variables including measures of neighbourhood disadvantage, covariates, and each mental health outcome. Due to differences in the numbers of children with missing data across the three outcomes, this resulted in slightly different numbers of children included in each analysis (externalising difficulties: n = 180,657; internalising difficulties: n = 180,654; competence: n = 176,408). Detailed information on missing data is presented in Supplementary Materials (Table S2).

### 2.13. Ethics Approval

Approvals from the Royal Children’s Hospital (RCH) Human Research Ethics Committee (HREC) (#30016), RMIT University HREC (#20749), AEDC data custodians (180130C), and the authorised data linkage agency (AIFS) were obtained for this project.

## 3. Results

### 3.1. AEDC-BE Cohort Characteristics

Demographic characteristics of the children included in the AEDC-BE capital cities cohort are presented in Table 1. The capital cities cohort was 48.7% female. Over one-quarter (28.0%) of the children in this cohort had a multilingual language background (English-proficient or -emerging), a slightly higher proportion than in the overall 2015 AEDC national cohort (21.5%) [60].

A lower proportion of Aboriginal and Torres Strait Islander children (2.8%) lived in these eight capital cities, as compared with the national cohort (5.7%) which includes regional towns and remote areas [60]. A substantial proportion of children in this cohort were from university-educated families (40.9% from families in which mothers achieved a bachelor’s degree or higher) and from Australia’s least disadvantaged neighbourhoods (27.4% residing in the least disadvantaged neighbourhood quintile). This is higher than the proportion of children who live in the least disadvantaged neighbourhoods in the national cohort (19.7%) [60].

### 3.2. Inequities in Access to Public Open Space

Overall, access to any type of public open space (irrespective of quality) was high: 95% of the cohort had access to a public open space within 800 m from home along the road network, and over 75% had access within 400 m (Table 1); however, several subgroups of children were disproportionately represented in the groups without access (i.e., ‘none’ within 800 m, 400 m). For example, despite making up only 2.8% of the overall capital cities cohort, Aboriginal and Torres Strait Islander children accounted for 3.9% of children without access to any public open space within 800 m. As shown in Table 1, there were also several subgroups of children who were disproportionately underrepresented in the optimal (i.e., child friendly) public open space exposure group. This was apparent at both the 800 m and 400 m distance thresholds. For example, despite making up 11.3% of the capital cities cohort, children in the lowest maternal education group made up only 8.9% and 8.6% of the children with access to child friendly public open space within 800 m and 400 m, respectively. Similarly, 15.9% of the children in the capital cities cohort lived in the most disadvantaged neighbourhoods, but these children represented only 13.5% (800 m) and 12.5% (400 m) of the child friendly public open space exposure group. These results illustrate systemic inequities in the provision of public resources that support health and wellbeing.

Descriptive statistics computed separately for each capital city (see Supplementary Materials, Tables S3–S10) showed that the distribution of children from different demographics and maternal education groupings across each of the public open space exposure groups (no public open space, other public open space, child friendly public open space) was similar to the national-level descriptive statistics shown in Table 1. An exception to this was the relationship between neighbourhood disadvantage and the public open space exposure groups. For example, in Adelaide, Canberra, and Melbourne, children from the least disadvantaged neighbourhoods (quintile 5) were over-represented in the group with no access to public open space within 800 m (the reference group), whereas in Hobart and Brisbane, children living in the most disadvantaged neighbourhoods (quintile 1) were over-represented in the group with no access to public open space.

**Figure 1:**
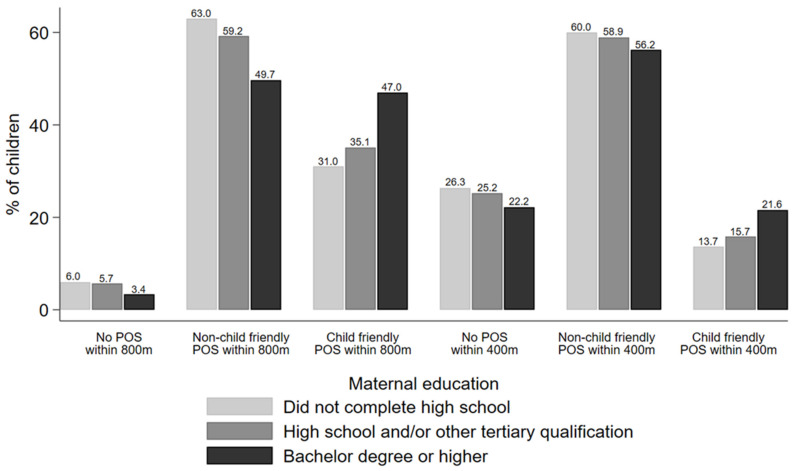
Percentages of children within each maternal education subgroup who have access to public open space within 800 m and 400 m from home along the road network. Children with missing data on maternal education (n = 16,662) not shown. POS: public open space. Child friendly POS defined as having both playground and public toilet nearby; non-child friendly POS defined as those lacking either/both of these child friendly features.

Furthermore, as shown in Figure 1, the percentages of children with access to public open space (and the presence of child friendly features) varied across the maternal education subgroups; this gradient in access was most striking when examining access to child friendly public open space. For example, in terms of the percentage of children with access to a child friendly public open space within 800 m from home, there was a 12- and 16-percentage point difference between children in the highest maternal education group, and those in the middle and lowest maternal education groups, respectively. A similar gradient was evident at the 400 m distance threshold.

**Figure 2:**
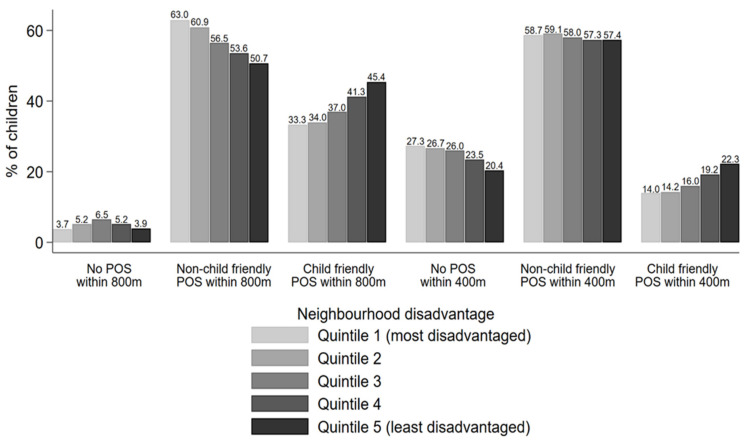
Percentages of children within each maternal education subgroup who have access to public open space (none, non-child friendly, child friendly) within 800 m and 400 m from home along the road network). Children with missing data on neighbourhood disadvantage (n = 829) not shown. POS: public open space. Child friendly POS defined as having both playground and public toilet nearby; non-child friendly POS defined as those lacking either/both of these child friendly features.

Similarly, Figure 2 shows a gradient in access to child friendly public open space across quintiles of neighbourhood disadvantage. For example, in terms of access to a child friendly public open space within 800 m from home, there was a 12 percentage-point difference between the least (45.4%) and most (33.3%) disadvantaged neighbourhoods, with the latter having poorer access. These differences were not just between the most and least disadvantaged neighbourhoods, but rather, they were illustrative of a gradient cutting across all five quintiles, where each quintile of disadvantage corresponded with a step down in the percentage of children with access to child friendly public open space, at both the 800 m and 400 m distance thresholds. When examining access within 800 m, a large percentage of children living in the most disadvantaged neighbourhoods had access to some type of public open space, but this was more likely to be non-child friendly types of open space.

### 3.3. Associations between Public Open Space and Child Mental Health

**Figure 3:**
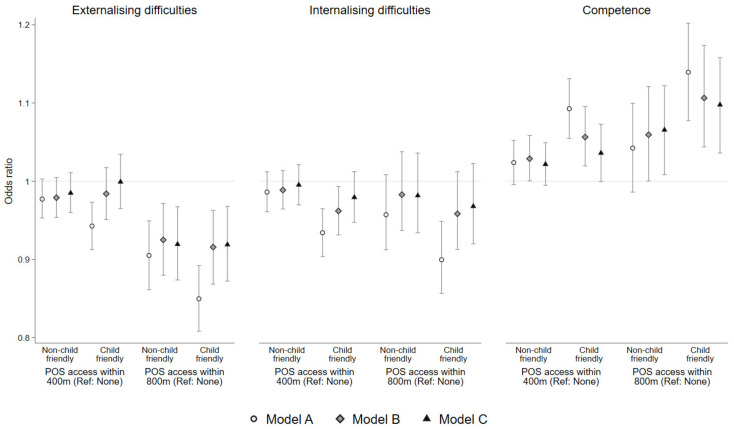
Unadjusted (Model A) and adjusted (Models B and C) associations (odds ratios and 95% credible intervals) between public open space exposures and children’s odds of demonstrating externalising difficulties, internalising difficulties, and high competence. Model A: unadjusted associations. Model B: adjusted for child’s sex, language background, and English proficiency, Aboriginal and Torres Strait Islander background, additional health and education needs, and maternal education. Model C: adjusted for child- and family-level demographics (Model B) plus neighbourhood disadvantage. For all models, the reference category for public open space exposures was the no access (none) group.

Results from the two-level unadjusted logistic regression model (Model A) and the two-level models adjusted for child- and family level-characteristics only (Model B) and child-, family-, and neighbourhood-level factors (Model C) are presented for the full capital cities cohort in Table 2 and Figure 3. Unadjusted associations were in the hypothesised direction for all exposures and outcomes (i.e., having better public open space access was associated with lower odds of difficulties and higher odds of competence). Furthermore, across all public open space exposures and outcomes, unadjusted associations indicated that children with access to a child friendly public open space had lower odds of difficulties and higher odds of competence than those who had access to non-child friendly types of public open space. These associations are attenuated somewhat in Models B and C, which adjust for different confounder sets. For each of the three mental health outcomes, Models B and C yielded similar results, meaning that the theoretical assumptions about the nature of the causal relationship between neighbourhood disadvantage and public open space access did not have a material impact on the findings.

Across all three outcomes examined, and all three adjustment sets, larger associations were observed between children’s mental health and public open space access within 800 m, rather than the 400 m distance threshold. Unadjusted associations indicated that children with access to public open space had better mental health (lower odds of internalising and externalising difficulties, and higher odds of competence), and the magnitude of association was larger if the public open space had child friendly features. Unadjusted associations were smallest in magnitude for children’s internalising difficulties.

In general, the associations attenuated after adjusting for child-, family-, and neighbourhood-level factors (Models B and C). For externalising and internalising difficulties, after adjustment, the association with child friendly public open space was similar to the association with non-child friendly public open space. For children’s competence, after adjusting for confounders in Models B and C, results were more suggestive of an additional benefit of child friendly features: the magnitude of association for child friendly public open space remained slightly larger than the magnitude of association for non-child friendly public open space within 800 m. This patterning was similar, though less distinct, at the 400 m distance threshold.

### 3.4. Effect Modification by Maternal Education

Table 3 and Table 4 show the results of the effect modification analysis (i.e., testing whether associations between public open space exposures and child mental health outcomes were different in each of the maternal education subgroups). These results are presented in a tabular format developed by Knol and VanderWeele for presenting the results of investigations into effect modification [67].

Overall, there was weak-to-moderate evidence of effect modification by maternal education on the association between children’s mental health and access to public open space. Looking across all three mental health outcomes and each of the public open space exposure measures, differences in the magnitude or direction of association were most evident for children in the lowest maternal education group than for those in the middle group, and when examining access to child friendly public open space (rather than access to non-child friendly public open space). In some instances, associations appeared larger for children in the lowest education group, whereas in other instances, the associations were smaller or in the opposite-than-anticipated direction for this group of children.

Regarding children’s externalising difficulties, stratum-specific adjusted odds ratios provided weak-to-moderate evidence that children in the lowest maternal education group may especially benefit (i.e., larger magnitude of protective association) from living within 800 m of child friendly public open space, compared with their peers from the middle and high education groups who had child friendly public open space access. This aligned with the hypothesised patterning of effect modification. For example, children in the lowest maternal education group had 17% lower odds of demonstrating externalising difficulties (stratum-specific AOR: 0.83; 95% CrI: 0.73–0.94) if they had access to a child friendly public open space within 800 m, versus no access to public open space. In the middle and highest maternal education groups, the association appeared to be smaller, with children having 6% (stratum-specific AOR: 0.94; 95% CrI: 0.88–1.00) and 7% (stratum-specific AOR: 0.93; 95% CrI: 0.85–1.02) reduced odds of externalising difficulties, respectively, if they had access to a child friendly public open space within 800 m, compared with no access to public open space. The measure of effect modification for the lowest maternal education group with access to child friendly public open space provided weak-to-moderate evidence to support this (ratio of odds ratios: 0.89; 95% CrI: 0.76–1.03). A somewhat similar pattern was observed for externalising difficulties and public open space access at the 400 m distance threshold; however, stratum-specific measures of association (and their credible intervals) between externalising difficulties and each of the public open space exposures at 400 m were consistent with no association within all maternal education groups.

In terms of children’s internalising difficulties and competence, there was weaker evidence of effect modification, and this patterning was in the opposite-than-anticipated direction for internalising difficulties. Children in the lowest maternal education group were observed to have higher likelihood of internalising difficulties if they lived within 800 m of child friendly public open space (stratum-specific AOR: 1.10; 95% CrI: 0.95–1.25) compared with those without access to public open space, whereas their peers in the middle and highest education groups had a lower likelihood of demonstrating internalising difficulties if they had access, compared with those who did not (stratum-specific AORs: 0.97 and 0.91, respectively). The measure of effect modification for the lowest maternal education group with access to child friendly public open space within 800 m provided weak-to-moderate evidence to support this (ratio of odds ratios: 1.19; 95% CrI: 1.01–1.41) and similar patterning was observed for public open space access within 400 m. For children’s competence, results showed that children in the middle and highest maternal education groups who had access to either type of public open space within 800 m had higher odds of demonstrating high competence than those who did not have access to any public open space (stratum-specific AORs ranging from 1.06 to 1.12); however, children in the lowest maternal education group who lived within 800 m of these spaces did not appear to receive the same benefit (stratum-specific AORs of 0.98 for both non-child friendly and child friendly public open space). The measure of effect modification for the lowest maternal education group with access to child friendly public open space provided weak-to-moderate evidence to support this patterning (ratio of odds ratios: 0.87; 95% CrI: 0.73–1.03). At the 400 m distance threshold, however, results generally appeared similar across the maternal education groups (consistent with no association), with small associations observed in the middle education group only (AORs: 1.04 and 1.06 for non-child friendly and child friendly public open space access, respectively).

## 4. Discussion

This study examined associations between multiple measures of public open space access, including quality-related measures, and a comprehensive assessment of children’s mental health (internalising difficulties, externalising difficulties, and competence) across a population of over 150,000 school-entry aged children residing in Australia’s eight capital cities. We also examined how access to, and quality of, public open space is distributed along subgroups of children.

### 4.1. Inequities in Access to Public Open Space across Capital Cities Cohort

Our results indicate that access to child friendly public open space is not randomly distributed, but follows a social gradient, cutting across both family socioeconomic position (maternal education) and neighbourhood disadvantage quintiles. With each step up in neighbourhood disadvantage (or each step down in maternal education), the percentage of children with access to child friendly (i.e., having both a playground and public toilet nearby) public open space within the specified distances was incrementally lower. This inequitable distribution aligns with previous Australian findings [31]. Furthermore, we found evidence that Aboriginal and Torres Strait Islander children were over-represented in the group without access to child friendly public open space within 800 m. These differences represent systemic inequities in provision of health-supporting resources. Addressing these inequities should be prioritised as part of Australia’s commitments to the UN Convention on the Rights of the Child and the Sustainable Development Goals, which call for access to green and public spaces for all (Goal 11, target 7) [68].

It is likely that the social gradient in public open space access and quality relates to the spatial distribution of disadvantage across cities, which is influenced by housing (un)affordability and processes like gentrification [69]. For example, in Australia’s capital cities, there is a shortage of affordable housing located in the inner ring; as a result, families facing socioeconomic disadvantage tend to be overrepresented in two types of neighbourhoods: ageing, middle ring higher-density neighbourhoods, and low-density outer suburban neighbourhoods [70]. Public open space provision may also drive increases in property values and housing costs, leading to gentrification and the displacement of families facing socioeconomic disadvantage [71,72]. Understanding how these processes are unfolding in Australian cities will be critical to informing strategies to improve urban liveability. For example, some advocate for making cities ‘just green enough’ without exacerbating gentrification [33]. Recent scholarship on the liveability of Australia’s cities calls for action across several domains, including improving housing affordability as well as access to high-quality public open space [44].

### 4.2. Public Open Space Access as a Protective and Promotive Factor

At a national level, we found evidence of protective associations between public open space access and the development of mental health difficulties in this capital cities cohort. Our analysis found that access to public open space was associated with lower odds of externalising difficulties, and marginally lower odds of internalising difficulties, in the early years. This aligns with findings from previous studies conducted in Australia, as well as North American and European studies [23,73,74,75]. The national pooled estimates indicated a similar benefit for public open space with child friendly features and public open space of other types. Other Australian studies focusing on the city of Perth have observed that access to public open space of higher quality or attractiveness appears more important to children’s early social development than access to public open space in general, whereas no association was detected with emotional development [24,37]. The difference in findings may be due to the use of outcomes which capture somewhat different aspects of mental health difficulties (i.e., vulnerability in social competence and emotional maturity) or the different measure of public open space quality (park attractiveness score).

We also found some evidence of an association between public open space access and children’s competence, suggesting this exposure may also be a promotive factor (i.e., promoting optimal mental health, beyond the prevention of difficulties). Nationally, the association was larger in magnitude for public open space with child friendly features than for those lacking these features. This finding was consistent with the theoretical framing of this study, which hypothesised that access to child friendly public open space with features that support extended use by families provides a key neighbourhood opportunity for skill-building and socialisation during early years. It adds to the growing evidence base concerning the importance of public open space for building children’s competence [26], and the importance of public open space quality, in particular [23,24], for promoting mental health during early years.

Our results showed larger magnitudes of association between child mental health and public open space access when access was defined using the larger distance threshold of 800 m. Previous studies testing associations between health outcomes and the built environment using multiple distance thresholds have also noted larger effect sizes for larger distances [76]. The stratified analysis also showed greater consistency of associations when access was defined using 800 m as a threshold. This supports the notion that families in Australia’s capital cities may be willing to travel further distances, even when children are young, to reach more desirable destinations.

### 4.3. For Whom Does Access Matter Most? Associations within Maternal Education Groups

Overall, we found weak-to-moderate evidence of effect modification across the maternal education groups, which suggests that access matters similarly for all children, but more research in this area is needed to clarify the nature of these associations. The results here partly align with findings from two previous studies from Lithuania and the United Kingdom, respectively, which observed that for children in the lowest maternal education group, living closer to public open space [77] or in areas with a higher percentage of natural space [78] was associated with fewer externalising difficulties; however, these same studies found that these measures of public open space were also associated with more prosocial behaviour, in contrast with the results here. Our results suggested that children in the lowest maternal education subgroup did not benefit as much from public open space access in terms of their competence, as compared with children in the middle and high maternal education subgroups. Our results also contrast with a previous study from the United Kingdom that found that children in the lowest socioeconomic group, living in areas with a higher percentage of green space, was associated with fewer internalising difficulties [79], whereas the results here found opposite-than-anticipated association with higher odds of internalising difficulties for this subgroup of children.

These conflicting findings could be due to several potential reasons, including different outcome measures used (e.g., prosocial behaviour, versus our multidimensional measure of competence), the studies being conducted in different countries, or other contextual factors. There may be additional quality features of public open space, beyond those measured here, that matter for building competence (e.g., the quality of play equipment and extent to which it facilitates risk-taking and creative play) and reducing odds of internalising difficulties (e.g., natural elements such as trees and water features). It is also likely that other neighbourhood-level factors (e.g., perceptions of neighbourhood safety) intersect with public open space quality to influence families’ likelihood of using these spaces, as well as the level of social activity in these spaces (and by extension, the opportunities for social interaction and skill-building). Finally, the divergent findings could be the result of unmeasured confounding in the present study, given that we were not able to account for other aspects of socioeconomic position (e.g., income) which may impact on where families live, independently of maternal education.

### 4.4. Similarities and Differences between Distinct Capital Cities

Although the national results were illustrative of a social gradient in public open space access across neighbourhood disadvantage quintiles, we found that in some cities the relationship between neighbourhood disadvantage and public open space access was less straightforward. It is possible that other contextual factors, such as the urban form, location of (un)affordable housing in relation to the city centre, or city size also play into the neighbourhood disadvantage—public open space access relationship in different ways for each capital city.

### 4.5. Strengths of This Study

A major strength of this study is its use of a unique population dataset, the AEDC-BE dataset, which is inclusive of all children entering the first year of school in Australia’s capital cities, reducing potential for selection bias which is particularly critical given the focus on children from disadvantaged backgrounds who are otherwise typically underrepresented in samples. The AEDC-BE dataset includes valuable information about children’s socioeconomic and demographic backgrounds and neighbourhood environments. To our knowledge, this is the first time such a linked population dataset has been made available internationally. Furthermore, the use of validated, theoretically established measures of competence, externalising, and internalising difficulties [7,10,35,36] was a key strength to reduce measurement bias.

### 4.6. Limitations

The results of this study should be viewed in the light of some limitations. We theorised a causal relationship between access to child friendly public open space and early childhood mental health, but there are limitations to the extent to which our results can provide evidence of this. For example, this study was a cross-sectional analysis (i.e., data taken from a single time point), so we cannot rule out reverse causation (where families’ places of residence are determined in part by their child’s experience of mental health difficulties or competence, which can impact on family resources available for housing, for example via treatment costs and caregivers’ capacity to work). Despite the inclusion of important demographics, our analysis was not able to account for certain family-level factors (e.g., caregivers’ mental health, parenting styles and practices), caregivers’ perceptions of neighbourhood safety, or information on families’ actual usage of public open space. In addition, maternal education is only one aspect of family socioeconomic position, which is a multidimensional construct and includes other factors like household income [80]. These unmeasured factors—and those pertaining to family socioeconomic positions in particular—are likely to be important sources of confounding of the relationship between public open space access and child mental health. It is possible that our results overestimate the true magnitude of association, given the inequitable patterning of public open space access and known impacts of socioeconomic position on mental health. This study used an administrative geographic unit (SA1) to measure the concept of neighbourhood disadvantage, which may not necessarily align with time spent in the neighbourhood (for further discussion of this issue, see works by Kwan [81] and Laatikainen and colleagues [82]). This may introduce bias through measurement error, leading to an underestimation of the true magnitude of confounding by neighbourhood disadvantage. Finally, our definition of public open space quality was based on qualitative research conducted with families with young children across Australia [17], who stressed these two features (playground and public toilet) were critical determinants of families’ usage of these spaces; however, there are likely other quality features that further encourage use of these public open spaces, such as vegetation and tree coverage, amenities, and safety. Future research could more deeply explore the features that encourage or determine families’ use of these spaces using both quantitative and qualitative techniques to inform more child- and family-centred urban planning and design guidelines.

### 4.7. Implications and Directions for Future Research

Our findings represent a first look at how public open space access and child friendliness across Australia’s capital cities relates to children’s mental health in their early years. Although our findings in the unadjusted analyses were consistent with the hypothesised direction across all outcomes and for both public open space exposure groups, the stratified analyses revealed some variation across cities (particularly around how public open space relates to neighbourhood disadvantage) that requires further unpacking. Several directions for future research are needed to enhance understandings of how public open space relates to young children’s mental health. First, future quantitative analyses drawing on longitudinal data, ideally with detailed information on families’ socioeconomic circumstances, will be critical to unpacking the causal relationships between disadvantage, public open space access and quality, and children’s mental health in distinct Australian cities as well as internationally. As mentioned earlier, research on factors influencing public open space use and quality aspects relevant to families with young children will be important for triangulating findings. In particular, qualitative studies are needed that focus on the neighbourhood features that might promote young children’s competence, specifically, and especially among families experiencing disadvantage. Lastly, urban scholarship unpacking the processes of gentrification and housing (un)affordability, and how this relates to the distribution of child friendly public open space in diverse Australian cities and towns, will be important to informing urban planning and policy responses and redressing inequities.

## 5. Conclusions

This study examined access to and quality of public open space and young children’s mental health across eight Australian capital cities using a unique population linked dataset, the Australian Early Development Census—Built Environment. A gradient in access to child friendly public open space was detected, with children facing disadvantage having incrementally poorer access with each step down the socioeconomic ladder. This is of concern, given that children with access to public open space close to home were also found to have a lower likelihood of demonstrating externalising and internalising difficulties and higher likelihood of demonstrating competence. Furthermore, we found weak-to-moderate evidence that these associations might be different in children from lower socioeconomic backgrounds. Further research is needed to clarify these potential differences, including qualitative research with children and families into the factors influencing public open space use and the quality features most salient for building children’s competence. Addressing inequities in child mental health requires multisectoral action. Together, findings from this study suggest that reducing inequities in access to neighbourhood resources, including child friendly public open space, should be included as part of a multifaceted strategy to improve mental health in early childhood.

## Figures and Tables

**Table 1:** AEDC-BE capital cities cohort characteristics and demographic characteristics of children in each public open space access group (none, non-child friendly, or child friendly public open space within 800 m, 400 m from child’s home).

		POS within 800 m			POS within 400 m		
	Full Cohort	None	Non-Child Friendly	Child Friendly	None	Non-Child Friendly	Child Friendly
All children, N (% of capital cities cohort)	N = 199,200 (100%)	N = 9627(4.8%)	N = 111,588(56.0%)	N = 77,985(39.1%)	N = 48,111(24.2%)	N = 115,538(58.0%)	N = 35,551(17.8%)
Sex							
% Female	48.7	48.4	48.5	48.9	48.5	48.7	48.8
Language background and English proficiency *							
% English-only	71.9	85.6	70.9	71.7	73.4	71.0	72.9
% Multilingual, English-proficient	24.6	12.5	25.5	24.9	23.3	25.5	23.8
% Multilingual, English-emerging	3.4	1.9	3.6	3.4	3.3	3.5	3.2
Aboriginal and Torres Strait Islander							
% Aboriginal and Torres Strait Islander	2.8	3.9	3.0	2.4	3.0	2.8	2.4
Additional health or education needs							
% Yes	4.6	4.2	4.8	4.3	4.4	4.7	4.4
Maternal education (highest level achieved) *							
% Bachelor’s degree or higher	40.9	28.7	36.4	48.6	37.6	39.7	49.1
% High school and/or other post-school qualification	47.8	57.1	50.8	42.5	50.0	48.6	42.3
% Did not complete high school	11.3	14.2	12.8	8.9	12.4	11.7	8.6
Neighbourhood disadvantage quintile *							
% Quintile 5 (least disadvantaged)	27.4	22.1	24.8	31.8	23.1	27.1	34.3
% Quintile 4	22.4	23.9	21.4	23.5	21.7	22.1	24.1
% Quintile 3	18.5	24.9	18.7	17.5	19.9	18.5	16.6
% Quintile 2	15.8	16.9	17.2	13.7	17.4	16.1	12.6
% Quintile 1 (most disadvantaged)	15.9	12.2	17.9	13.5	17.9	16.1	12.5

Notes: POS: public open space. * Indicates measures with missing data for some children (see Table S2 in Supplementary Materials). Percentages were calculated for all children with data available (i.e., children with missing outcome data were not excluded from descriptive statistics). Child friendly POS defined as having both playground and public toilet nearby; non-child friendly POS defined as those lacking either/both of these child friendly features.

**Table 2:** Associations between public open space access (non-child friendly and child friendly) and children’s odds of mental health outcomes (externalising, internalising, competence) across Australia’s eight capital cities.

	Model A		Model B		Model C	
	OR	95% CrI	AOR	95% CrI	AOR	95% CrI
***Externalising***						
Non-child friendly POS (800 m)	0.91	(0.86, 0.95)	0.93	(0.88, 0.97)	0.92	(0.87, 0.97)
Child friendly POS (800 m)	0.85	(0.81, 0.89)	0.92	(0.87, 0.96)	0.92	(0.87, 0.97)
Non-child friendly POS (400 m)	0.98	(0.95, 1.00)	0.98	(0.95, 1.00)	0.98	(0.96, 1.01)
Child friendly POS (400 m)	0.94	(0.91, 0.97)	0.98	(0.95, 1.02)	1.00	(0.97, 1.03)
***Internalising***						
Non-child friendly POS (800 m)	0.96	(0.91, 1.01)	0.98	(0.94, 1.04)	0.98	(0.93, 1.04)
Child friendly POS (800 m)	0.90	(0.86, 0.95)	0.96	(0.91, 1.01)	0.97	(0.92, 1.02)
Non-child friendly POS (400 m)	0.99	(0.96, 1.01)	0.99	(0.96, 1.01)	1.00	(0.97, 1.02)
Child friendly POS (400 m)	0.93	(0.90, 0.96)	0.96	(0.93, 0.99)	0.98	(0.95, 1.01)
***Competence***						
Non-child friendly POS (800 m)	1.04	(0.99, 1.10)	1.06	(1.00, 1.12)	1.07	(1.01, 1.12)
Child friendly POS (800 m)	1.14	(1.08, 1.20)	1.11	(1.04, 1.17)	1.10	(1.04, 1.16)
Non-child friendly POS (400 m)	1.02	(1.00, 1.05)	1.03	(1.00, 1.06)	1.02	(0.99, 1.05)
Child friendly POS (400 m)	1.09	(1.05, 1.13)	1.06	(1.02, 1.10)	1.04	(1.00, 1.07)

Notes: Reference group for all models is children in the ‘No access to POS’ group within 800 m, 400 m. Model A: Unadjusted associations. Model B: Adjusted for maternal education and child demographics (sex, Aboriginal, and Torres Strait Islander background, language background and proficiency, additional health and education needs) only. Model C: Adjusted for child- and family-level demographics (Model B) plus neighbourhood disadvantage. OR: unadjusted odds ratio. AOR: adjusted odds ratio. 95% CrI: 95% credible interval. POS: public open space.

**Table 3:** Results from the investigation of effect modification for measures of public open space within 800 m. Based on template developed by Knol and VanderWeele [67].

	No POS		Non-Child Friendly POS		Child Friendly POS		AOR (95% CI) within Each Maternal Education Stratum	
	N with/without Outcome	AOR (95% CrI)	N with/without Outcome	AOR (95% CrI)	N with/without Outcome	AOR (95% CI)	Non-Child Friendly POS vs. No POS	Child Friendly POS vs. No POS
***Externalising***								
Bachelor’s degree or higher	794/1,693	1.0 (Reference)	11,060/25,552	0.93 (0.86, 1.02)	10,327/24,376	0.93 (0.85, 1.02)	0.93 (0.86, 1.02)	0.93 (0.85, 1.02)
High school and/or other tertiary qualification	1925/3021	1.33 (1.21, 1.48)	19,077/32,076	1.23 (1.12, 1.34)	11,335/18,993	1.25 (1.14, 1.37)	0.92 (0.86, 0.98)	0.94 (0.88, 1.00)
Did not complete high school	573/657	1.70 (1.48, 1.95)	5619/7238	1.51 (1.37, 1.65)	2661/3680	1.40 (1.26, 1.55)	0.89 (0.79, 1.01)	0.83 (0.73, 0.94)
***Internalising***								
Bachelor’s degree or higher	801/1684	1.0 (Reference)	11,214/25,381	0.96 (0.87, 1.06)	10,253/24,444	0.91 (0.84, 1.01)	0.96 (0.87, 1.06)	0.91 (0.84, 1.01)
High school and/or other tertiary qualification	1771/3174	1.12 (1.01, 1.24)	17,964/33,169	1.08 (0.99, 1.20)	10,582/19,720	1.09 (0.99, 1.20)	0.97 (0.91, 1.03)	0.97 (0.91, 1.04)
Did not complete high school	485/743	1.21 (1.04, 1.41)	5223/7624	1.28 (1.16, 1.42)	2616/3716	1.32 (1.19, 1.47)	1.06 (0.93, 1.20)	1.09 (0.95, 1.24)
***Competence***								
Bachelor’s degree or higher	799/1644	1.0 (Reference)	12,042/23,766	1.06 (0.96, 1.16)	12,051/21,831	1.12 (1.02, 1.24)	1.06 (0.96, 1.16)	1.12 (1.02, 1.24)
High school and/or other tertiary qualification	1391/3491	0.81 (0.73, 0.91)	14,477/35,512	0.88 (0.80, 0.97)	8671/20,852	0.88 (0.80, 0.97)	1.08 (1.00, 1.16)	1.09 (1.01, 1.17)
Did not complete high school	289/924	0.71 (0.60, 0.83)	2856/9660	0.70 (0.63, 0.78)	1398/4754	0.69 (0.61, 0.77)	0.98 (0.86, 1.12)	0.98 (0.84, 1.13)

Notes: All models adjusted for child’s sex, language background and English proficiency, Aboriginal and Torres Strait Islander background, additional health and education needs, neighbourhood disadvantage. Adjusted odds ratios and credible intervals presented in the first three columns (titled ‘No POS,’ ‘Non-child friendly POS,’ and ‘Child friendly POS’ were calculated based on a single reference category: children whose mothers completed a bachelor’s degree or higher and who do not have access to public open space within 800 m from home. Adjusted odds ratios presented in the last two columns (titled ‘AOR (95% CrI) within each education level’) were calculated separately for each maternal education group, with distinct reference categories (i.e., children who do not have access) for each maternal education group. AOR adjusted odds ratio. 95% CrI 95% credible interval. POS public open space. Measures of effect modification: all measures of effect modification were calculated as a ratio of odds ratios (multiplicative scale), with the denominator being the odds ratio for the highest maternal education group (bachelor’s degree or higher). *Measures of effect modification: externalising difficulties.* Non-child friendly POS and middle maternal education group (high school and/or other tertiary qualification): 0.99 (95% CrI: 0.88–1.09). Child friendly POS and middle maternal education group: 1.01 (95% CrI: 0.90–1.12). Non-child friendly POS and lowest maternal education group (did not complete high school): 0.95 (95% CrI: 0.82–1.10). Child friendly POS and lowest maternal education group: 0.89 (95% CrI: 0.76–1.03). *Measures of effect modification: internalising difficulties.* Non-child friendly POS and middle maternal education group: 1.02 (95% CrI: 0.91–1.13). Child friendly POS and middle maternal education group: 1.07 (95% CrI: 0.95–1.19). Non-child friendly POS and lowest maternal education group: 1.11 (95% CrI: 0.95–1.30). Child friendly POS and lowest maternal education group: 1.19 (95% CrI: 1.01–1.41). *Measures of effect modification: competence.* Non-child friendly POS and middle maternal education group: 1.03 (95% CrI: 0.91–1.15). Child friendly POS and middle maternal education group: 0.97 (95% CrI: 0.86–1.09). Non-child friendly POS and lowest maternal education group: 0.94 (95% CrI: 0.79–1.12). Child friendly POS and lowest maternal education group: 0.87 (95% CrI: 0.73–1.03).

**Table 4:** Results from investigation of effect modification for measures of public open space within 400 m. Based on template developed by Knol and VanderWeele [67].

	No POS		Non-Child Friendly POS		Child Friendly POS		AOR (95% CI) within Each Education Stratum	
	N with/without Outcome	AOR (95% CI)	N with/without Outcome	AOR (95% CI)	N with/without Outcome	AOR (95% CI)	Non-Child Friendly POS vs. No POS	Child Friendly POS vs. No POS
***Externalising***								
Bachelor’s degree or higher	4934/11,485	1.0 (Reference)	12,451/29,016	1.00 (0.96, 1.04)	4796/11,120	1.02 (0.97, 1.07)	1.00 (0.96, 1.04)	1.02 (0.97, 1.07)
High school and/or other tertiary qualification	8261/13,585	1.35 (1.29, 1.42)	18,937/31,946	1.31 (1.26, 1.37)	5139/8559	1.35 (1.28, 1.42)	0.97 (0.94, 1.01)	1.00 (0.95, 1.05)
Did not complete high school	2373/3020	1.60 (1.49, 1.71)	5316/6925	1.61 (1.52, 1.70)	1164/1630	1.48 (1.35, 1.62)	1.01 (0.94, 1.08)	0.93 (0.83, 1.03)
***Internalising***								
Bachelor’s degree or higher	4990/11,421	1.0 (Reference)	12,606/28,850	1.01 (0.97, 1.05)	4672/11,238	0.97 (0.92, 1.02)	1.01 (0.97, 1.05)	0.97 (0.92, 1.02)
High school and/or other tertiary qualification	7725/14,112	1.17 (1.12, 1.22)	17,849/33,011	1.15 (1.11, 1.20)	4743/8940	1.14 (1.09, 1.20)	0.99 (0.95, 1.02)	0.98 (0.93, 1.03)
Did not complete high school	2194/3191	1.35 (1.26, 1.45)	4956/7275	1.36 (1.29, 1.43)	1174/1617	1.44 (1.32, 1.57)	1.00 (0.94, 1.08)	1.07 (0.97, 1.17)
***Competence***								
Bachelor’s degree or higher	5521/10,529	1.0 (Reference)	13,916/26,632	1.01 (0.97, 1.05)	5455/10,080	1.01 (0.97, 1.05)	1.01 (0.97, 1.05)	1.01 (0.97, 1.05)
High school and/or other tertiary qualification	6090/15,298	0.79 (0.75, 0.82)	14,466/35,185	0.82 (0.79, 0.86)	3983/9372	0.84 (0.79, 0.89)	1.04 (1.00, 1.08)	1.06 (1.01, 1.12)
Did not complete high school	1195/4078	0.65 (0.60, 0.71)	2722/9176	0.65 (0.61, 0.69)	626/2084	0.65 (0.58, 0.71)	1.00 (0.91, 1.08)	0.99 (0.88, 1.11)

Notes: All models adjusted for child’s sex, language background and English proficiency, Aboriginal and Torres Strait Islander background, additional health and education needs, neighbourhood disadvantage. Adjusted odds ratios and credible intervals presented in the first three columns (titled ‘No POS,’ ‘Non-child friendly POS,’ and ‘Child friendly POS’ were calculated based on a single reference category: children whose mothers completed a bachelor’s degree or higher and who do not have access to public open space within 400 m from home. Adjusted odds ratios presented in the last two columns (titled ‘AOR (95% CrI) within each education level’) were calculated separately for each maternal education group, with distinct reference categories (i.e., children who do not have access) for each maternal education group. AOR adjusted odds ratio. 95% CrI 95% credible interval. POS public open space. Measures of effect modification: all measures of effect modification were calculated as a ratio of odds ratios (multiplicative scale), with the denominator being the odds ratio for the highest maternal education group (bachelor’s degree or higher). *Measures of effect modification: externalising difficulties.* Non-child friendly POS and middle maternal education group (high school and/or other tertiary qualification): 0.97 (95% CrI: 0.92–1.03). Child friendly POS and middle maternal education group: 0.98 (95% CrI: 0.91–1.05). Non-child friendly POS and lowest maternal education group (Did not complete high school): 1.01 (95% CrI: 0.93–1.09). Child friendly POS and lowest maternal education group: 0.91 (95% CrI: 0.81–1.02). *Measures of effect modification: internalising difficulties.* Non-child friendly POS and middle maternal education group: 0.98 (95% CrI: 0.93–1.03). Child friendly POS and middle maternal education group: 1.02 (95% CrI: 0.95–1.09). Non-child friendly POS and lowest maternal education group: 1.00 (95% CrI: 0.92–1.08). Child friendly POS and lowest maternal education group: 1.10 (95% CrI: 0.99–1.23). *Measures of effect modification: competence.* Non-child friendly POS and middle maternal education group: 1.03 (95% CrI: 0.98–1.09). Child friendly POS and middle maternal education group: 1.05 (95% CrI: 0.97–1.12). Non-child friendly POS and lowest maternal education group: 0.99 (95% CrI: 0.90–1.09). Child friendly POS and lowest maternal education group: 0.97 (95% CrI: 0.85–1.10).

## Data Availability

The linked AEDC-BE dataset may be available for research purposes pending appropriate data use approvals from the Human Research Ethics Committee, data custodians, and research team. For more information, please contact: hannah.badland@rmit.edu.au.

## Supplementary Materials

The following supporting information can be downloaded at: https://www.mdpi.com/article/10.3390/ijerph19116780/s1, Figure S1: Directed acyclic graph (DAG) used to develop the analysis plan, Table S1: Description of public open space access and quality measures, based on guidance developed by Lamb and colleagues [52], Table S2: Missing data across study variables, Table S3: Adelaide cohort characteristics and demographic characteristics of children in each public open space access group (none, non-child friendly, or child friendly public open space within 800 m, 400 m from child’s home), Table S4: Brisbane cohort characteristics and demo-graphic characteristics of children in each public open space access group (none, non-child friendly, or child friendly public open space within 800 m, 400 m from child’s home), Table S5: Canberra cohort characteristics and demographic characteristics of children in each public open space access group (none, non-child friendly, or child friendly public open space within 800 m, 400 m from child’s home), Table S6: Darwin cohort characteristics and demographic characteristics of children in each public open space access group (none, non-child friendly, or child friendly public open space within 800 m, 400 m from child’s home), Table S7: Hobart cohort characteristics and demographic characteristics of children in each public open space access group (none, non-child friendly, or child friendly public open space within 800 m, 400 m from child’s home), Table S8: Melbourne cohort characteristics and demographic characteristics of children in each public open space access group (none, non-child friendly, or child friendly public open space within 800 m, 400 m from child’s home), Table S9: Perth cohort characteristics and demographic characteristics of children in each public open space access group (none, non-child friendly, or child friendly public open space within 800 m, 400 m from child’s home), Table S10: Sydney cohort characteristics and demographic characteristics of children in each public open space access group (none, non-child friendly, or child friendly public open space within 800 m, 400 m from child’s home).
