# Supplementary material for: Access to and Quality of Neighbourhood Public Open Space and Children’s Mental Health Outcomes: Evidence from Population Linked Data across Eight Australian Capital Cities
Source: Int J Environ Res Public Health. 2022 Jun 1;19(11):6780. doi: 10.3390/ijerph19116780 (PMC9180559; doi:10.3390/ijerph19116780)
Supplement: Supplementary file 1 [file ijerph-19-06780-s001.zip › ijerph-1677630-supplementary.pdf]

Table S1: Description of public open space access and quality measures, based on guidance developed by Lamb and colleagues [1].

			Subtypes		Spatial data source(s)						
	**Measure**	**Description**	**Included**	**Excluded**	**Provider**	**Year**	**Cost**	**Spatial extent and characteristics**	**Limitations**	**Ground-truthing?**	**Rationale for use**
1	Distance to closest public open space (any type) (metres)	Each child's home address was matched to a sample point along the road network (mean match distance of 3.5 metres in capital cities cohort). This measure represents the distance in metres from the sample point to the closest entry point along the boundary of any public open space along the walkable road network (calculated using methods described by Higgs and colleagues [2]).	Open space with at least some publicly accessible land (area of public land greater than 0 hectares) tagged by OpenStreetMap contributors as: leisure (is not null), beach (is not null), public squares (place= 'square'), other pedestrian areas (highway= 'pedestrian'); with land-use tagged as any of the following: common, conservation, forest, garden, leisure, park, recreation_ground, sport, trees, village_green, winter_sports, wood, dog_park, nature_reserve, off_leash, sports_centre, riverbank, beach.	Non-public areas of open space: e.g., schools, golf courses, sports stadiums, zoos, theme parks, cemeteries, military areas, agricultural areas, forestry areas.	OpenStreet Map	2018	$0	National (OpenStreetMap has global coverage)	Although generally very consistent, community contributed data may contain some deviations from the community guidelines for tagging public open space. Proxy entry points may not exactly match real entry points.	In-person ground truthing not feasible given the large geographic scope. Instead, desktop validity checks were conducted using local geospatial databases (e.g., public open space data maintained by Victorian Environmental Assessment Council and Brisbane council) and satellite imagery (e.g., Google Maps satellite imagery of open space).	Nationally consistent coverage; regularly archived data; community guidelines provide a standard for contributing to OpenStreetMap. Community contributions updated in real-time.
2	Distance to closest child friendly public open	Same as measure 1, plus additional criterion that public open space is 'child friendly.'	Same as measure 1 above, with additional restrictions applied: public open space tagged as having a toilet	Same as measure 1 above; in addition public open	OpenStreet Map	2018	$0	National (OpenStreetMap has global coverage)	Although generally very consistent, community	In-person ground truthing not feasible given the large	Nationally consistent coverage; regularly archived data; community guidelines provide a

	Measure	Description	Subtypes: Included	Subtypes: Excluded	Spatial data source(s): Provider	Year	Cost	Spatial extent and characteristics	Limitations	Ground-truthing?	Rationale for use
	space (metres)	Child friendly public open space was defined as having both a playground and a nearby toilet.	and playground nearby (i.e., a toilet located within 100 metres Euclidean distance from the boundary of the public open space).	space was excluded if not tagged as having a playground or nearby toilet.					contributed data may contain some deviations from the community guidelines for tagging public open space. Proxy entry points may not exactly match real entry points.	geographic scope. Instead, desktop validity checks were conducted using local geospatial databases (e.g., public open space data maintained by Victorian Environmental Assessment Council and Brisbane council) and satellite imagery (e.g., Google Maps satellite imagery of open space).	standard for contributing to OpenStreetMap. Community contributions updated in real-time.
3	Access to public open space (none, non-child friendly, or child friendly) within 800m	Derived from measures 1 and 2 above. Access to 'non-child friendly' or 'child friendly' if public open space was located within 800 metres (road network buffer).	See as measures 1 and 2 above.								
4	Access to public open space (none,	Derived from measures 1 and 2 above. Access to	See measures 1 and 2 above.								

Measure	Description	Subtypes: Included	Subtypes: Excluded	Spatial data source(s): Provider	Year	Cost	Spatial extent and characteristics	Limitations	Ground-truthing?	Rationale for use
non-child friendly, or child friendly) within 400m	'non-child friendly' or 'child friendly' if public open space was located within 400 metres (road network buffer).									

Table S2: Missing data across study variables.

Variable	Missing data N (%)
Sex	0 (0%)
Language background and English proficiency	529 (0.3%)
Indigenous status	0 (0%)
Additional health and education needs	0 (0%)
Maternal education	16662 (8.4%)
Neighbourhood disadvantage	829 (0.4%)
Public open space access within 800m	0 (0%)
Public open space access within 400m	0 (0%)
Externalising difficulties	1367 (0.7%)
Internalising difficulties	1471 (0.7%)
Competence	6055 (3.0%)
Average missing across all study variables	1.23%

Table S3: Adelaide cohort characteristics and demographic characteristics of children in each public open space access group (none, non-child friendly, or child friendly public open space within 800 metres, 400 metres from child's home).

		POS within 800m			POS within 400m		
	**Full cohort**	**None**	**Non-child friendly**	**Child friendly**	**None**	**Non-child friendly**	**Child friendly**
All children, N (% of Adelaide cohort)	N=15,157 (100.0)	N=893 (5.9)	N=8,617 (56.9)	N=5,647 (37.3)	N=3,785 (25.0)	N=9,018 (59.5)	N=2,354 (15.5)
Sex							
% Female	47.9	49.0	47.9	47.8	47.9	48.1	47.2
Language background and English proficiency*							
% English-only	79.4	88.1	79.1	78.5	79.5	79.2	80.1
% Multilingual, English-proficient	17.8	9.3	18.1	18.8	17.3	18.2	17.4
% Multilingual, English-emerging	2.7	2.6	2.8	2.7	3.2	2.6	2.5
Aboriginal and Torres Strait Islander							
% Aboriginal and Torres Strait Islander	3.2	1.6	3.4	3.2	3.0	3.4	2.9
Additional health or education needs							
% Yes	5.3	4.6	5.7	4.8	4.4	5.7	5.1
Maternal education (highest level achieved)*							
% Bachelor's degree or higher	34.8	28.1	31.1	41.4	34.1	33.1	42.0
% Year 12 and/or other post-school qualification	51.3	56.7	53.5	47.2	52.6	51.9	46.8
% Year 11 or less	13.9	15.1	15.5	11.4	13.2	15.0	11.1
Neighbourhood disadvantage quintile*							
% Quintile 5 (least disadvantaged)	16.1	21.3	15.4	16.3	15.2	16.2	17.0
% Quintile 4	20.8	21.0	17.8	25.4	19.7	19.2	28.8
% Quintile 3	19.8	27.4	17.8	21.6	21.8	18.0	23.3
% Quintile 2	21.7	18.2	23.5	19.4	23.2	22.4	16.2
% Quintile 1 (most disadvantaged)	21.7	12.1	25.6	17.3	20.2	24.2	14.7

Note: *POS:* public open space. *Indicates measures with missing data for some children (see Table S2). Percentages were calculated for all children with data available (i.e., children with missing outcome data were not excluded from descriptive statistics). Child friendly POS defined as having both playground and public toilet nearby; non-child friendly POS defined as those lacking either/both of these child friendly features.

Table S4: Brisbane cohort characteristics and demographic characteristics of children in each public open space access group (none, non-child friendly, or child friendly public open space within 800 metres, 400 metres from child's home).

		POS within 800m			POS within 400m		
	**Full cohort**	**None**	**Non-child friendly**	**Child friendly**	**None**	**Non-child friendly**	**Child friendly**
All children, N (% of Brisbane cohort)	N=31,741 (100.0)	N=2,318 (7.3)	N=16,704 (52.6)	N=12,719 (40.1)	N=7,786 (24.5)	N=17,942 (56.5)	N=6,013 (18.9)
Sex							
% Female	48.9	48.1	48.9	49.1	48.6	49.2	48.3
Language background and English proficiency*							
% English-only	84.3	95.2	82.5	84.6	87.7	82.5	85.3
% Multilingual, English-proficient	13.3	4.1	14.7	13.0	10.4	14.8	12.3
% Multilingual, English-emerging	2.5	0.7	2.8	2.3	1.9	2.7	2.4
Aboriginal and Torres Strait Islander							
% Aboriginal and Torres Strait Islander	4.5	5.7	4.5	4.1	5.2	4.3	4.0
Additional health or education needs							
% Yes	4.4	3.7	4.6	4.3	4.0	4.5	4.5
Maternal education (highest level achieved)*							
% Bachelor's degree or higher	37.0	24.8	33.8	43.3	33.3	36.4	43.6
% Year 12 and/or other post-school qualification	51.5	60.6	53.8	46.7	54.2	52.0	46.4
% Year 11 or less	11.5	14.6	12.3	10.0	12.5	11.7	10.0
Neighbourhood disadvantage quintile*							
% Quintile 5 (least disadvantaged)	26.6	16.1	24.0	31.8	21.2	26.4	34.1
% Quintile 4	21.7	19.3	20.8	23.4	21.5	21.5	22.8
% Quintile 3	19.4	28.8	20.8	15.8	22.7	19.4	15.1
% Quintile 2	16.5	21.5	17.8	13.8	18.8	16.6	12.9
% Quintile 1 (most disadvantaged)	15.8	14.2	16.5	15.2	15.8	16.1	15.1

Note: *POS:* public open space. *Indicates measures with missing data for some children (see Table S2). Percentages were calculated for all children with data available (i.e., children with missing outcome data were not excluded from descriptive statistics). Child friendly POS defined as having both playground and public toilet nearby; non-child friendly POS defined as those lacking either/both of these child friendly features.

Table S5: Canberra cohort characteristics and demographic characteristics of children in each public open space access group (none, non-child friendly, or child friendly public open space within 800 metres, 400 metres from child's home).

		POS within 800m			POS within 400m		
	**Full cohort**	**None**	**Non-child friendly**	**Child friendly**	**None**	**Non-child friendly**	**Child friendly**
All children, N (% of Canberra cohort)	N=5,421 (100.0)	N=106 (2.0)	N=3,796 (70.9)	N=1,519 (28.0)	N=530 (9.8)	N=3,972 (73.3)	N=919 (17.0)
Sex							
% Female	49.2	46.2	48.6	50.9	46.4	49.6	49.0
Language background and English proficiency*							
% English-only	77.5	87.7	78.3	74.7	84.8	77.3	73.7
% Multilingual, English-proficient	19.3	11.3	18.8	21.2	13.4	19.4	22.2
% Multilingual, English-emerging	3.2	0.9	2.9	4.1	1.7	3.2	4.0
Aboriginal and Torres Strait Islander							
% Aboriginal and Torres Strait Islander	2.7	9.4	2.4	3.0	4.3	2.3	3.5
Additional health or education needs							
% Yes	4.3	4.7	4.2	4.5	4.0	4.3	4.5
Maternal education (highest level achieved)*							
% Bachelor's degree or higher	53.4	28.9	52.7	56.9	43.2	55.1	51.5
% Year 12 and/or other post-school qualification	40.5	56.7	41.4	37.2	46.6	39.5	42.0
% Year 11 or less	6.1	14.4	5.9	5.8	10.1	5.5	6.5
Neighbourhood disadvantage quintile*							
% Quintile 5 (least disadvantaged)	56.7	63.2	57.9	53.2	45.1	58.3	56.3
% Quintile 4	26.5	4.7	24.9	32.0	43.5	21.4	39.0
% Quintile 3	10.4	32.1	9.6	10.9	10.0	11.9	3.8
% Quintile 2	4.6	0.0	5.2	3.6	1.4	6.0	0.5
% Quintile 1 (most disadvantaged)	1.8	0.0	2.5	0.3	0.0	2.4	0.3

Note: *POS:* public open space. *Indicates measures with missing data for some children (see Table S2). Percentages were calculated for all children with data available (i.e., children with missing outcome data were not excluded from descriptive statistics). Child friendly POS defined as having both playground and public toilet nearby; non-child friendly POS defined as those lacking either/both of these child friendly features.

Table S6: Darwin cohort characteristics and demographic characteristics of children in each public open space access group (none, non-child friendly, or child friendly public open space within 800 metres, 400 metres from child's home).

		POS within 800m			POS within 400m		
	**Full cohort**	**None**	**Non-child friendly**	**Child friendly**	**None**	**Non-child friendly**	**Child friendly**
All children, N (% of Darwin cohort)	N=1,905 (100.0)	N=347 (18.2)	N=906 (47.6)	N=652 (34.2)	N=624 (32.8)	N=994 (52.2)	N=287 (15.1)
Sex							
% Female	49.1	49.3	48.8	49.5	51.3	47.7	49.5
Language background and English proficiency*							
% English-only	74.6	85.8	74.7	68.4	80.7	71.5	72.0
% Multilingual, English-proficient	22.0	13.0	21.8	27.0	16.9	24.6	24.1
% Multilingual, English-emerging	3.4	1.2	3.5	4.6	2.4	4.0	3.8
Aboriginal and Torres Strait Islander							
% Aboriginal and Torres Strait Islander	16.8	15.3	19.6	13.7	16.8	17.5	14.3
Additional health or education needs							
% Yes	5.0	6.1	5.1	4.3	6.1	4.8	3.1
Maternal education (highest level achieved)*							
% Bachelor's degree or higher	31.8	29.9	25.8	41.3	31.8	29.5	39.6
% Year 12 and/or other post-school qualification	54.0	56.8	57.8	47.1	53.8	55.8	48.3
% Year 11 or less	14.2	13.3	16.3	11.6	14.4	14.7	12.1
Neighbourhood disadvantage quintile*							
% Quintile 5 (least disadvantaged)	26.3	23.1	29.4	23.8	21.9	29.6	25.1
% Quintile 4	26.1	26.2	19.5	35.2	28.8	21.8	34.8
% Quintile 3	24.3	31.7	19.0	27.6	27.0	22.5	24.4
% Quintile 2	15.8	16.7	19.9	9.7	16.1	16.9	11.5
% Quintile 1 (most disadvantaged)	7.5	2.3	12.1	3.8	6.3	9.2	4.3

Note: *POS:* public open space. *Indicates measures with missing data for some children (see Table S2). Percentages were calculated for all children with data available (i.e., children with missing outcome data were not excluded from descriptive statistics). Child friendly POS defined as having both playground and public toilet nearby; non-child friendly POS defined as those lacking either/both of these child friendly features.

Table S7: Hobart cohort characteristics and demographic characteristics of children in each public open space access group (none, non-child friendly, or child-friendly public open space within 800 metres, 400 metres from child's home).

		POS within 800m			POS within 400m		
	**Full cohort**	**None**	**Non-child friendly**	**Child friendly**	**None**	**Non-child friendly**	**Child friendly**
All children, N (% of Hobart cohort)	N= 2,842 (100.0)	N=442 (15.6)	N=1,344 (47.3)	N=1,056 (37.2)	N=1,351 (47.5)	N=1,002 (35.3)	N=489 (17.2)
Sex							
% Female	48.3	47.5	46.5	50.9	48.6	46.9	50.3
Language background and English proficiency*							
% English-only	93.8	98.2	95.1	90.3	94.1	94.1	92.4
% Multilingual, English-proficient	5.1	1.6	3.9	8.0	4.8	4.6	6.7
% Multilingual, English-emerging	1.1	0.2	1.0	1.7	1.1	1.3	0.8
Aboriginal and Torres Strait Islander							
% Aboriginal and Torres Strait Islander	6.9	8.8	7.8	5.0	7.8	7.0	4.5
Additional health or education needs							
% Yes	3.7	3.4	4.1	3.4	4.0	3.5	3.5
Maternal education (highest level achieved)*							
% Bachelor's degree or higher	30.9	16.0	26.6	42.9	24.4	33.2	43.9
% Year 12 and/or other post-school qualification	49.6	60.7	52.4	41.1	54.1	47.4	41.4
% Year 11 or less	19.6	23.4	21.0	16.0	21.4	19.4	14.6
Neighbourhood disadvantage quintile*							
% Quintile 5 (least disadvantaged)	14.3	14.5	14.8	13.4	12.1	17.1	14.3
% Quintile 4	21.4	11.1	18.5	29.4	16.1	23.2	32.3
% Quintile 3	20.6	20.1	18.9	23.0	20.9	19.7	21.7
% Quintile 2	16.5	23.1	17.7	12.4	19.2	14.1	14.3
% Quintile 1 (most disadvantaged)	27.2	31.2	30.1	21.8	31.7	25.9	17.4

Note: *POS:* public open space. *Indicates measures with missing data for some children (see Table S2). Percentages were calculated for all children with data available (i.e., children with missing outcome data were not excluded from descriptive statistics). Child friendly POS defined as having both playground and public toilet nearby; non-child friendly POS defined as those lacking either/both of these child friendly features.

Table S8: Melbourne cohort characteristics and demographic characteristics of children in each public open space access group (none, non-child friendly, or child friendly public open space within 800 metres, 400 metres from child's home).

		POS within 800m			POS within 400m		
	**Full cohort**	**None**	**Non-child friendly**	**Child friendly**	**None**	**Non-child friendly**	**Child friendly**
All children, N (% of Melbourne cohort)	N= 53,985 (100.0)	N=2,119 (3.9)	N=32,054 (59.4)	N=19,812 (36.7)	N=13,612 (25.2)	N=32,030 (59.3)	N=8,343 (15.5)
Sex							
% Female	48.6	48.4	48.3	49.2	48.9	48.4	48.8
Language background and English proficiency*							
% English-only	70.7	84.2	69.0	72.1	74.1	68.9	72.5
% Multilingual, English-proficient	25.6	13.3	27.1	24.4	22.6	27.2	24.0
% Multilingual, English-emerging	3.7	2.5	3.9	3.6	3.3	3.9	3.6
Aboriginal and Torres Strait Islander							
% Aboriginal and Torres Strait Islander	0.9	0.8	0.9	1.0	1.0	0.9	0.9
Additional health or education needs							
% Yes	5.1	5.2	5.4	4.7	5.0	5.2	4.8
Maternal education (highest level achieved)*							
% Bachelor's degree or higher	42.9	32.8	38.9	50.4	41.7	41.4	50.6
% Year 12 and/or other post-school qualification	46.6	55.1	49.6	40.8	47.5	47.8	40.8
% Year 11 or less	10.5	12.1	11.4	8.7	10.8	10.8	8.7
Neighbourhood disadvantage quintile*							
% Quintile 5 (least disadvantaged)	24.1	27.3	21.8	27.4	23.2	23.2	28.5
% Quintile 4	25.6	29.1	24.8	26.4	25.9	25.2	26.6
% Quintile 3	21.1	21.2	21.7	20.0	21.1	21.5	19.3
% Quintile 2	15.2	12.8	17.2	12.4	15.2	16.1	12.1
% Quintile 1 (most disadvantaged)	14.1	9.6	14.5	13.9	14.7	14.0	13.5

Note: *POS:* public open space. *Indicates measures with missing data for some children (see Table S2). Percentages were calculated for all children with data available (i.e., children with missing outcome data were not excluded from descriptive statistics). Child friendly POS defined as having both playground and public toilet nearby; non-child friendly POS defined as those lacking either/both of these child friendly features.

Table S9: Perth cohort characteristics and demographic characteristics of children in each public open space access group (none, non-child friendly, or child friendly public open space within 800 metres, 400 metres from child's home).

		POS within 800m			POS within 400m		
	**Full cohort**	**None**	**Non-child friendly**	**Child friendly**	**None**	**Non-child friendly**	**Child friendly**
All children, N (% of Perth cohort)	N=25,866 (100.0)	N=895 (3.5)	N=17,385 (67.2)	N=7,586 (29.3)	N=5,248 (20.3)	N=17,354 (67.1)	N=3,264 (12.6)
Sex							
% Female	48.4	47.5	48.3	48.7	48.4	48.4	48.3
Language background and English proficiency*							
% English-only	78.5	87.2	78.8	76.6	79.2	78.5	77.4
% Multilingual, English-proficient	18.9	11.6	18.5	20.8	18.4	18.9	20.0
% Multilingual, English-emerging	2.6	1.1	2.6	2.6	2.4	2.6	2.6
Aboriginal and Torres Strait Islander							
% Aboriginal and Torres Strait Islander	3.4	4.0	3.9	2.3	3.8	3.6	2.1
Additional health or education needs							
% Yes	3.5	2.7	3.7	3.2	3.4	3.6	3.4
Maternal education (highest level achieved)*							
% Bachelor's degree or higher	38.2	24.0	34.0	49.5	36.6	36.6	49.3
% Year 12 and/or other post-school qualification	49.0	60.3	51.6	41.9	49.6	50.1	42.2
% Year 11 or less	12.7	15.7	14.4	8.6	13.8	13.2	8.5
Neighbourhood disadvantage quintile*							
% Quintile 5 (least disadvantaged)	31.8	22.6	29.6	38.1	27.3	32.1	38.0
% Quintile 4	25.1	27.8	24.2	27.0	22.7	25.3	28.1
% Quintile 3	18.0	27.2	17.7	17.6	20.3	17.4	17.8
% Quintile 2	15.9	14.4	17.3	13.0	19.7	15.5	12.2
% Quintile 1 (most disadvantaged)	9.1	8.0	11.1	4.4	10.0	9.7	4.0

Note: *POS:* public open space. *Indicates measures with missing data for some children (see Table S2). Percentages were calculated for all children with data available (i.e., children with missing outcome data were not excluded from descriptive statistics). Child friendly POS defined as having both playground and public toilet nearby; non-child friendly POS defined as those lacking either/both of these child friendly features.

Table S10: Sydney cohort characteristics and demographic characteristics of children in each public open space access group (none, non-child friendly, or child friendly public open space within 800 metres, 400 metres from child's home).

		POS within 800m			POS within 400m		
	**Full cohort**	**None**	**Non-child friendly**	**Child friendly**	**None**	**Non-child friendly**	**Child friendly**
All children, N (% of Sydney cohort)	N=62,283 (100.0)	N=2,507 (4.0)	N=30,782 (49.4)	N=28,994 (46.6)	N=15,175 (24.4)	N=33,226 (53.4)	N=13,882 (22.3)
Sex							
% Female	48.8	48.8	49.0	48.6	48.1	48.9	49.3
Language background and English proficiency*							
% English-only	60.6	74.4	57.8	62.3	59.5	59.3	64.9
% Multilingual, English-proficient	35.1	22.9	37.6	33.6	35.8	36.3	31.4
% Multilingual, English-emerging	4.3	2.8	4.6	4.1	4.7	4.4	3.7
Aboriginal and Torres Strait Islander							
% Aboriginal and Torres Strait Islander	2.6	3.1	3.1	2.0	2.5	2.9	2.1
Additional health or education needs							
% Yes	4.5	4.1	4.6	4.3	4.2	4.6	4.4
Maternal education (highest level achieved)*							
% Bachelor's degree or higher	43.1	32.3	36.7	50.7	38.2	41.6	51.9
% Year 12 and/or other post-school qualification	46.1	54.2	50.1	41.3	49.4	46.9	40.6
% Year 11 or less	10.8	13.5	13.2	8.0	12.3	11.5	7.5
Neighbourhood disadvantage quintile*							
% Quintile 5 (least disadvantaged)	30.0	23.0	24.9	36.0	24.8	28.4	39.4
% Quintile 4	18.7	26.1	17.3	19.5	17.6	18.6	20.0
% Quintile 3	16.1	22.2	16.1	15.5	16.6	16.5	14.6
% Quintile 2	15.4	16.0	16.5	14.3	16.9	15.8	13.0
% Quintile 1 (most disadvantaged)	19.9	12.7	25.2	14.8	24.0	20.8	13.1

Note: *POS:* public open space. *Indicates measures with missing data for some children (see Table S2). Percentages were calculated for all children with data available (i.e., children with missing outcome data were not excluded from descriptive statistics). Child friendly POS defined as having both playground and public toilet nearby; non-child friendly POS defined as those lacking either/both of these child friendly features.

RMIT Classification: Trusted

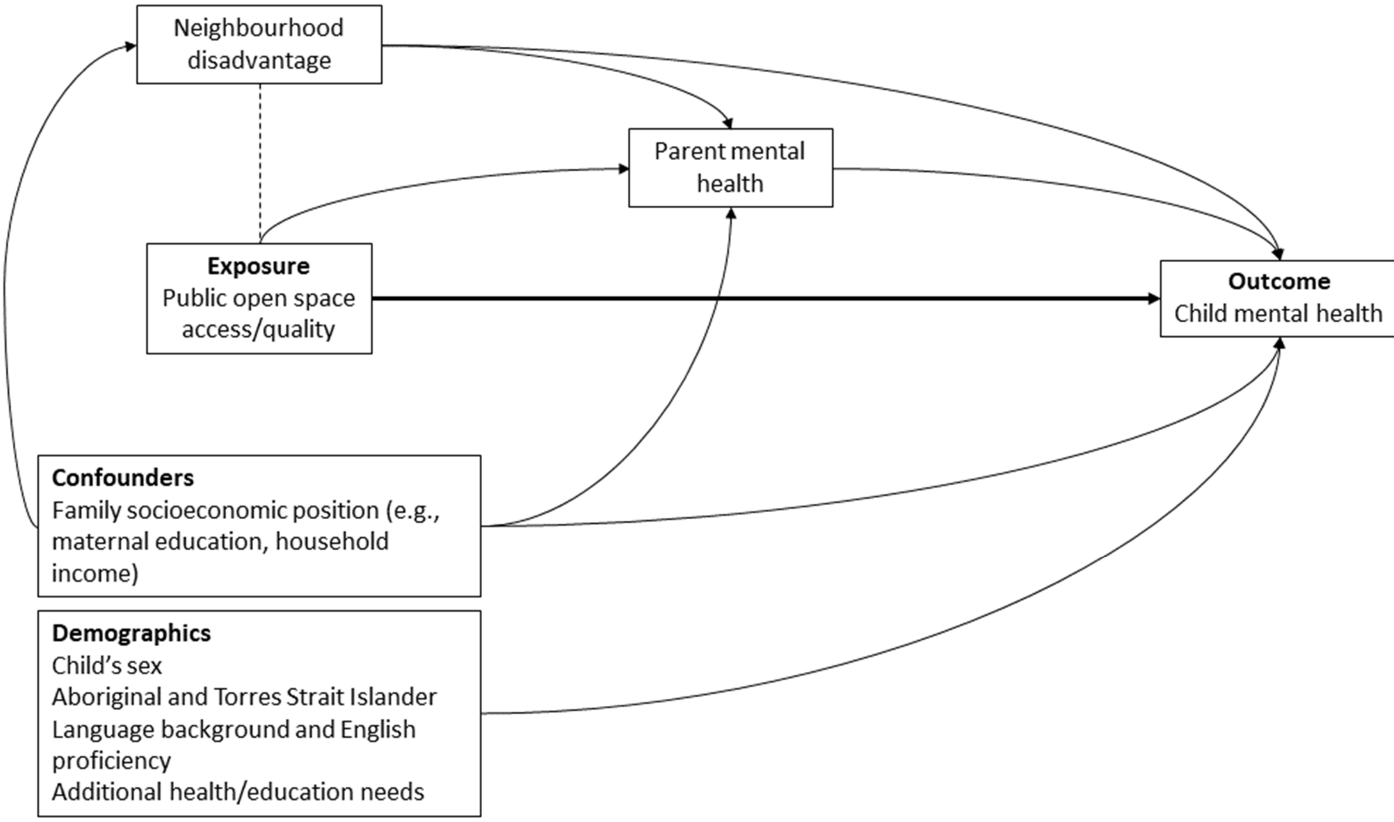

Figure S1: Directed acyclic graph (DAG) used to develop the analysis plan.

References

1. Higgs, C.; Badland, H.; Simons, K.; Knibbs, L.; Giles-Corti, B. The Urban Liveability Index: Developing a policy-relevant urban liveability composite measure and evaluating associations with transport mode choice. *Int. J. Health Geogr.* **2019**, *18*, 1-25.
2. Lamb, K.E.; Mavoa, S.; Coffee, N.T.; Parker, K.; Richardson, E.A.; Thornton, L.E. Public open space exposure measures in Australian health research: A critical review of the literature. *Geogr. Res.* **2019**, *57*, 67–83. https://doi.org/10.1111/1745-5871.12325.
